# 

**Published:** 1881-08

**Authors:** 


### CONTENTS FOR AUGUST, 1881.

ORIGINAL COMMUNICATIONS.

ART. I.—Lectures on the Treatment of Diseases of the Liver, Spleen and Intestines. By Prof. E. L. SCHURLY, M. D. Reported by HUGO ERICHSEN .......... 443
ART. II.—A Plea for the more frequent use of the Obstetric Forceps. By ROGER MARVIN GRISWOLD, M. D .......... 454
ART. III.—Responsibilities of Medical Colleges. By W. R. MONROE, M. D .......... 461
ART. IV.—Nitrous Oxide Gas. By LAURENCE TURNBULL, M. D .......... 464

EDITORIAL DEPARTMENT.

Alexis St. Martin .......... 473
Water and Sand Bags .......... 474
Seats for Shop Girls .......... 474
Bonwille's Method of Rapid Breathing .......... 475
Odor Mortis .......... 475
Summer, Malaria, &c .......... 477
Birth and Death Rate in Large Cities .......... 477
Diagnosis of Gastric Cancer .......... 478
Etiquette .......... 478
The Woman's Hospital .......... 479
The Food Adulteration Law .......... 480
Liquor Acidi Phosphorici .......... 480
Delegates to Medical Convention in London .......... 480
Annual Meeting of American Dental Association .......... 481
National Dental Association .......... 484

BIBLIOGRAPHICAL DEPARTMENT.

Index-Catalogue of Library of Surgeon-General's Office, Vol. 2 .......... 486
A Practical Treatise on Impotence, Sterility, &c .......... 486
The Mid-Continent .......... 487
Tenotony in Treatment of Congenital Club Foot .......... 487
The Arkansaw Doctor .......... 487
The Peoria Medical Monthly .......... 487
The Southern Medical Reformer .......... 488
N. Y. College of Dentistry .......... 488
THERAPEUTIC PEARLS AT RANDOM STRUNG .......... 488–489
SELECTIONS .......... 490-499

POPULAR SCIENCE DEPARTMENT.

Domestic Hygiene .......... 500
Food Adulteration Law .......... 508
HYMENEAL .......... 512
NECROLOGICAL .......... 513

